# Combined ultrasonic aspiration and saline-linked radiofrequency precoagulation: a step toward bloodless liver resection without the need of liver inflow occlusion: analysis of 313 consecutive patients

**DOI:** 10.1186/1477-7819-12-357

**Published:** 2014-11-25

**Authors:** Evangelos Felekouras, Athanasios Petrou, Kyriakos Neofytou, Alexandros Giakoustidis, Jessamy Bagenal, Ferdinando Cananzi, Emmanouel Pikoulis, Satvinder Mudan

**Affiliations:** First Department of Surgery, University of Athens Medical School, Laikon Teaching Hospital, Αγ. Θωμά 17, Goudi, Athens, Greece; Nicosia Surgical Department, Division of Hepatobiliary Pancreatic Surgery, Nicosia General Hospital, Palaios Dromos Lefkosias - Lemesou, 215, 2029 Strovolos, Nicosia, Cyprus; Department of Academic Surgery, Upper GI/HPB Unit, Royal Marsden Hospital, Fulham Road, London, SW3 6JJ UK; The London Clinic, 20 Devonshire Pl, London, W1G 6BW UK; Severn School of Surgery, Bristol Heart Institute, Bristol, UK

**Keywords:** Liver resection, Blood loss, Pringle, Complications, Ultrasonic aspiration, Radiofrequency

## Abstract

**Background:**

Hemorrhage is undoubtedly one of the main factors contributing to morbidity and mortality in liver resections. Vascular occlusion techniques are effective in controlling intraoperative bleeding, but they cause liver damage due to ischemia. We evaluated the effectiveness and safety of using a combined technique for hepatic parenchymal transection without liver inflow occlusion.

**Methods:**

Three hundred and thirteen consecutive patients who underwent liver resection in four hepato-pancreato-biliary units. Hepatic parenchymal transection was carried out using a combined technique of saline-linked radiofrequency precoagulation and ultrasonic aspiration without liver inflow occlusion.

**Results:**

During the study period 114 minor and 199 major hepatic resections were performed. The mean amount of intraoperative blood loss was 377 ml (SD 335 ml, range 50 to 2,400 ml) and the blood transfusion rate was 10.5%. The median amount of blood loss during parenchymal transection and parenchymal transection time was 222 ml (SD 224 ml, range 40 to 2,100 ml) and 61 minutes (range 12 to 150 minutes) respectively. There were two postoperative deaths (0.6%). Complications occurred in 84 patients (26.8%) and most complications were minor.

**Conclusions:**

Combined technique of saline-linked radiofrequency ablation and ultrasonic aspiration for liver resection is a safe method for both major and minor liver resections. The method is associated with decreased blood loss, reduced postoperative morbidity, and minimal mortality rates. We believe that this combined technique is comparable to other techniques and should be considered as an alternative.

## Background

Hepatic resection is widely accepted as the only potential curative treatment for patients with a wide variety of liver conditions[1–5]. Hemorrhage is one of the main factors contributing to morbidity and mortality in major liver resections[6–11]. However, bleeding and subsequent blood transfusion may increase the recurrence rate and reduce the survival rate for malignant diseases[7, 12–14].

The concept of introducing bloodless techniques to facilitate surgical resection of liver tumors has stimulated hepatobiliary surgeons to experiment with new procedures. Initial techniques involved clamping of outflow (total vascular exclusion), and inflow vessels (Pringle maneuver), aiming to reduce bleeding during parenchymal transaction[15–17]. The Pringle maneuver, which involves the control of the hepatic vascular inflow by clamping the hepatoduodenal ligament, represents the most simple of these methods of vascular control[18]. This maneuver represents a valuable tool for managing intraoperative bleeding, but at the same time places the patient at a high risk of liver damage due to ischemic reperfusion syndrome and many other complications[18–24]. Consequently, the use of liver occlusion has reduced among liver surgeons but is still used in a few centers. One European survey revealed that 19% of surgeons apply the Pringle maneuver routinely[25].

The need to avoid the complications related to major vascular occlusions has resulted in the use of liver parenchymal transection without clamping techniques. Parenchymal transection tools such as the Cavitron ultrasonic surgical aspirator (CUSA®), saline-linked radiofrequency precoagulation, harmonic scalpel, bipolar scissors, Ligasure device, hydrodissectors, or monopolar floating ball, single or in combinations, are commonly used by experienced surgical teams and the published results are encouraging. The combination of new transection tools, concomitant anesthetic and critical care improvements have dramatically reduced morbidity and mortality rates.

We report the efficacy and complications of a combined technique for liver resection without liver inflow occlusion in 313 consecutive patients with primary or metastatic liver malignancies. This technique uses combinational Cavitron ultrasonic surgical aspirator (CUSA; ValleyLab, Boulder, CO, USA) and a saline-linked radiofrequency dissecting sealer (Aquamantys®).

## Methods

From September 2007, to March 2013, all patients who underwent liver resection for liver malignancies, with combined technique using CUSA**®** and Aquamantys**®** at the following 4 hepato-pancreato-biliary (HPB) units: 1) Department of Academic Surgery, Royal Marsden Hospital, London, UK, 2) The London Clinic Hospital, London, UK, 3) Nicosia Department of Surgery, Division of HPB Surgery, Nicosia General Hospital, Nicosia, Cyprus, 4) First Department of Surgery, University of Athens Medical School, Laikon Teaching Hospital, Athens, Greece have been included. During this period, this combined technique was used by three HPB surgeons (FE, PA, MS).

In accordance with international guidelines, all the patients underwent the necessary preoperative assessment of their disease, including spiral computed tomography, magnetic resonance imaging, and/or positron emission tomography.

Patient data was collected retrospectively and included demographic details, histological type and number of tumors, surgical procedure, overall operating time, parenchymal transection time, overall amount of intraoperative blood loss (IBL) and blood loss during parenchymal transaction (PTBL). Major and critical abnormalities of preoperative and postoperative liver function tests, intraoperative and postoperative complications, mortality rate, the length of hospital stay, and outcome were also recorded. Post hepatectomy liver failure was defined as the presence of serum bilirubin >50 μmol/l and prothrombin index <50% in the fifth postoperative day according to the ‘50-50 criteria’[26].

The resection time was defined as the time from the start of CUSA® to the completion of parenchymal transection. The overall blood loss was estimated by weight of the surgical swabs, the amount of blood in the suction system and the CUSA® suction container following the entire hepatectomy. Blood loss during liver parenchyma transection was recorded in a similar way. Blood was transfused accordingly to maintain the hematocrit above 28 to 30% perioperatively or if the patient developed hemodynamic instability as a result of blood loss.

This retrospective analysis of liver resection using a combination of CUSA® and Aquamantys® devices without the need for the Pringle maneuver focuses on the following outcomes; intraoperative blood loss , parenchymal transection blood loss, need for packed red blood cell (PRBC) transfusion, length of liver resection time and postoperative morbidity and 30-day mortality.

For each participating institution, the study was approved by the local ethics committee and Institutional Review Board.

### Operative technique

Incisions used were a modified right subcostal (J shape), bilateral subcostal, or Mercedes-like. Central venous pressure was maintained at a low level (usually below 5 mmHg during the parenchyma transaction) to avoid backflow bleeding and to facilitate better liver tissue manipulation. To obviate air embolism, the hepatic resection was performed with the patient in a 15-degree Trendelenburg position. Minimum accepted urine output was defined at 25 ml /hour. Blood pressure to achieve these demands was maintained by either volume or vasopressor drug infusion whenever necessary.

In all cases, after exploration of the abdominal cavity to exclude extrahepatic or peritoneal disease, operative ultrasound was used to define the tumor, to exclude preoperatively undetected lesions and to mark the plane for liver parenchymal transection. Subsequently, the liver was mobilized according to the size and site of the lesion to be resected.

For major hepatectomies the ipsilateral major hepatic veins were encircled with vessel loops. Additionally, when an anatomic resection was planned, hilar dissection was performed. The ipsilateral branch of the hepatic artery, portal vein, and common bile duct were encircled but not divided until the parenchymal dissection reached that point. Hilar dissection was not performed for non-anatomical hepatectomy.

**Figure 1:**
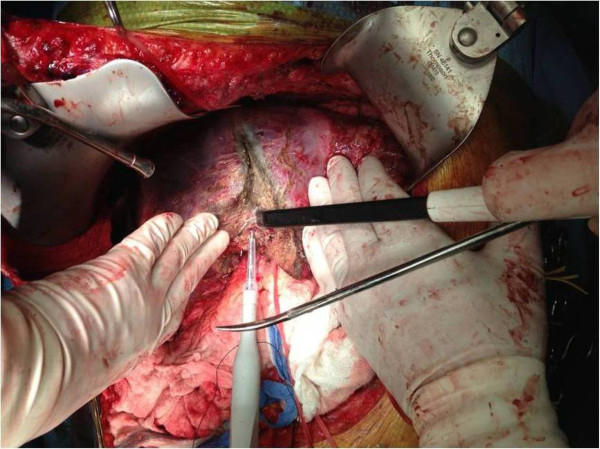
**Hepatic parenchymal transection using CUSA**® **and Aquamantys®.**

The liver parenchyma was divided by CUSA®. During parenchymal dissection the hepatic lobe or segment that was being resected was gently retracted by the assistant in order to separate the cut surfaces of the liver for optimal exposure (open book technique). A second surgeon held the Aquamantys**®** for coagulation and hemostasis (Figure 1). The Aquamantys® system, which is a bipolar sealer, delivers radiofrequency energy and saline simultaneously to the surgical field in order to provide hemostasis or sealing across exposed tissue. The radiofrequency energy heats tissues to around 100°C. As a result of the heat, the collagen in the blood vessel walls shrinks, causing hemostasis. At the same time the saline, which is delivered to the surgical field, prevents charring with eschar formation. The device was efficient in controlling the small bleeding vessels within the liver parenchyma. Larger vessels and large intrahepatic bile ducts were either ligated or clipped.

For major hepatectomies, the ipsilateral hepatic artery, portal vein branches and bile duct branches were ligated intrahepatically during parenchymal transection. In addition, for major hepatectomies, the major hepatic veins were either suture-ligated and divided or divided using endovascular staplers at the end of parenchymal transection. Drains were placed in all patients.

### Statistic analysis

We used SPSS statistical software, version 12 (SPSS Inc, Chicago, IL, USA), for data analysis. Common statistics were applied in order to estimate the significance of the results. Chi-square test, Mann–Whitney non-parametric test, and the Fischer’s exact test were used as appropriate. Differences were considered to be significant if *P* <0.05.

## Results

Three hundred and thirteen patients were included in the series (137 female and 176 males). The median age was 62 years (range 31 to 85 years). The median age among patients who underwent major liver resections (a resection of 3 segments or more) was 61 years and among patients undergoing minor liver resections (a resection of 2 or fewer segments) was 63 years (*P*-value 0.29). The majority of the patients (247 patients; 78.9%) had colorectal liver metastases (CRLM) and the rest had non-colorectal liver metastases (12 patients; 3.8%), hepatocellular carcinoma (24 patients; 7.7%), cholangiocarcinoma (25 patients; 8%), and gallbladder carcinoma (5 patients; 1.6%). The patient and tumor characteristics are given in Table 1.

**Table 1:** **Patient and tumor characteristics**

Characteristic	Finding			***P***-value
	Major Hepatectomy^a^	Minor Hepatectomy^b^	Total	
Age, mean (SD), (minimum-maximum) years	61 (10.74) (31 to 85)	62.55 (10.20) (37 to 82)	61.57 (10.56) (31 to 85)	0.29
Sex, F/M, n	88/111	49/65	137/176	0.832
Diagnosis, n				
Colorectal liver metastases	144	103	247	
Non-colorectal liver metastases	3	9	12	
Hepatocellular carcinoma	22	2	24	
Cholangiocarcinoma	25z	0	25	
Gallbladder carcinoma	5	0	5	
Total	199 (63.6%)	114 (36.4%)	313	
Number of lesions, one/more than one	82/117	63/51	145/168	0.016

^a^three or more segmentectomies.

^b^two or fewer segmentectomies or hepatic wedge resections-tumorectomies.

We performed 199 (63.58%) major hepatectomies and 114 (36.42%) minor hepatectomies (Table 2). One hundred and five (33.5%) hepatectomies were performed at the Royal Marsden Hospital, 80 (25.6%) at the The London Clinic Hospital, 48 (15.3%) at the Nicosia General Hospital and 80 (25.6%) in Laikon Teaching Hospital. The proportion of different tumor types (*P* <0.001) varied in each of the 4 HPB units; however the type of operation (major versus minor liver resection) regarding each tumor type was the same (*P* >0.05). Forty-two re-do hepatectomies were successfully performed (41 of recurrent CRLM and 1 of non-colorectal liver metastases). In addition, 41 of the patients suffering from CRLM underwent a synchronous liver resection in combination with colon resection. The liver lesions per patient rate, was statistically significant for the patients who underwent major liver resections compared to the patients who underwent minor liver resections (*P*-value 0.016) (Table 1).

**Table 2:** **Operation characteristics**

	Diagnosis					
	Colorectal liver metastases	Non-colorectal liver metastases	Hepatocellular carcinoma	Cholangiocarcinoma	Gallbladder carcinoma	Total
Major hepatectomy	144	3	22	25	5	199
Right hepatectomy (with or without combined wedge resection)	92	1	10	4	0	107
Left hepatectomy (with or without combined wedge resection)	36	2	10	3	0	51
Extended left hepatectomy	4	0	0	7	0	11
Extended right hepatectomy	12	0	2	11	5	30
Minor hepatectomy	103	9	2	0	0	114
Anatomical hepatectomy						
Right posterior hepatectomy	9	1	0	0	0	10
Left lateral hepatectomy	27	4	0	0	0	31
Bisegmentectomy	14	0	0	0	0	14
Segmentectomy	18	0	2	0	0	20
Non-anatomical hepatectomy						
Wedge resections (one or multiple)	35	4	0	0	0	39

In Tables 1 and2, it is clearly demonstrated that for patients suffering from hepatocellular carcinoma, hilar/intrahepatic cholangiocarcinoma, and gallbladder carcinoma that major hepatectomy as a treatment decision was more frequent compared to patients suffering from liver metastasis (96.29 versus 56.75%, *P*-value <0.001). When comparing the type and the extent of liver resection among patients with CRLM with those with non-colorectal liver metastases it is observed that major hepatectomies were more frequently performed in the first group of patients (42.1 versus 25%, *P* = 0.023).

The overall operative time (mean) was 210 minutes (minimum 90 minutes, maximum 550 minutes). The mean parenchymal transection time was 51 minutes (minimum 18 minutes, maximum 120 minutes). The operative time for parenchymal transection was affected due to the raw liver surface. Major hepatectomies were prolonged with mean parenchymal transection time 69 minutes and range 48 to 120 minutes compared to minor liver resections; mean 29 minutes, range 18 to 52 minutes (*P* <0.001).

The mean overall intraoperative blood loss was 377 ml (SD 335 ml, range 50 to 2,400 ml), and the mean blood loss during parenchymal transection was 223 ml (SD 224 ml, range 40 to 2,100 ml) (Table 3). Amongst the checked factors that potentially affect blood loss during parenchymal transection (gender, age, simultaneous procedures, extent of resection (major versus minor liver resection), re-do (second or third) hepatectomy, type of minor liver resection (anatomic versus non-anatomic liver resection)), the only factor that actually affected blood loss during parenchymal transection was the extent of liver resection (Table 4). In patients undergoing major hepatectomies, the mean blood loss during parenchyma transection was 274 ml (SD 264 ml), while in patients undergoing minor hepatectomies it was 131 ml (SD 55.06 ml) (274 ml versus 131 ml *P* <0.001). Although the PTBL was greater in non-anatomical liver resection than in anatomical minor liver resections (124 ml versus 145 ml), this difference was not statistically significant (*P* = 0.088). Contrary to the PTBL, the IBL was affected by more factors (Table 4). So, in patients undergoing major hepatectomies, the IBL was 436 ml, while in patients undergoing minor hepatectomies it was 273 ml (*P* <0.001). Moreover, in patients who underwent re-do liver resections and in patients who underwent combined colon and hepatic resection the IBL was higher (539 ml versus 352 ml, *P* <0.001 and 501 ml versus 358 ml, *P* = 0.001 respectively).

**Table 3:** **Blood loss**

	Type of operation			
	Major liver resection (199)	Minor liver resection (114)	Total (313)	***P***-value
Intraoperative blood loss, mean (SD), (range), ml	436.38 (346.76)	272.72 (286.08)	376.77 (334.92)	**< 0.001**
	(100 to 2,400)	(50 to 2,360)	(50 to 2,400)	
Blood loss during parenchymal transection, mean (SD), (range), ml	273.42 (264)	131.23 (55.06)	221.63 (223.66)	**< 0.001**
	(90 to 2,100)	(40 to 350)	(40 to 2,100)	
Blood transfusion, n, (%)	17 (8.5%)	16 (14%)	33 (10.5%)	0.128

**Table 4:** **Factors that potentially affect blood loss during parenchymal transaction and the overall amount of intraoperative blood loss**

Clinical factor	Number	Blood loss during parenchyma transaction mean (SD), ml	***P***-value	Overall amount of intraoperative blood loss mean (SD), ml	***P***-value
Overall	313	221.63 (223.66)		376.77 (334.92)	
Gender					
Female	137	220.8 (206.77)	0.789	358.91 (294.54)	0.374
Male	176	222.27 (236.55)		390.68 (363.49)	
Age (years)					
≤60	138	234 (258.84)	0.366	405.65 (398.17)	0.383
>60	175	211.9 (191.64)		354.01 (274.09)	
Concomitant procedures					
Yes	41	188.78 (75.14)	0.719	500.98 (434.44)	**0.001**
No	272	226.58 (237.84)		358.05 (314)	
Extent of resection					
Major	199	273.42 (264)	**< 0.001**	436.38 (346.76)	**< 0.001**
Minor	114	131.23 (55.06)		272.72 (286.08)	
Re-do hepatic resection					
Yes	42	247.14 (306.27)	0.228	539.29 (436.01)	**< 0.001**
No	271	217.68 (208.43)		351.59 (309.85)	
Type of minor liver resection					
Anatomic	75	123.87 (50.05)	0.088	278 (323.42)	0.56
Non-anatomic	39	145.38 (61.85)		262.56 (198.78)	

Thirty-three of our patients (10.5%) underwent blood transfusion either during the operation or during the short-term postoperative period. Seventeen of these patients underwent re-do liver resection or combined colon and hepatic resection for CRLM. Excluding the patients who underwent re-do liver resection or combined colon and hepatic resection for CRLM, the blood transfusion rate for the rest of the patients was reduced to 6.7%. This reduction of blood transfusion rate was predictable as the mean IBL, when we excluded these patients, decreased from 377 ml to 336 ml (*P* <0.001). Most of the transfusions were given during the operation because of heavy blood loss (up to 2,400 ml). The rest were given in the postoperative period.

Interestingly, while the IBL in major hepatectomies was higher than in minor hepatectomies (436 ml versus 273 ml) the blood transfusion rate was higher in minor liver resections although this difference was not statistically significant (8.5% versus 14%, *P* = 0.128). As is demonstrated in Table 1, within the group of patients with minor liver resection, the percentage of patients with CRLM was higher than the corresponding one within the group of patients with major liver resection (103 patients with CRLM/114 minor liver resections versus 144 patients with CRLM/199 major liver resections *P* <0.001). This corresponds to the fact that the highest percentage of patients within the minor liver resection group had received neoadjuvant chemotherapy when compared to the major liver resection group (92/114 versus 124/199, 80.7% versus 62.3%, *P* <0.001). This feature raises the possibility that the cause for higher transfusion rates in the minor liver resection group, despite the fact of a lower intraoperative blood loss, was the lower preoperative hematocrit as a result of neoadjuvant chemotherapy.

The length of hospital stay ranged from 3 to 42 days with a mean value of 6 days.

A total of 119 complications occurred in 84 patients (26.8%). Complications and their management are summarized in Table 5. Analysis of complications between major and minor liver resections revealed no statistical differences between outcomes (Table 5). According to the Clavien Classification of Surgical Complications, only 21 patients (6.7%) developed major complications (grade III and IV). Regarding the 16 patients (5.11%) who developed a bile leak, 7 of them had a biliary anastomosis in addition to a liver resection.

**Table 5:** **Postoperative complications and their management**

	Type of operation				Management
	Major liver resection (199)	Minor liver resection (114)	Total	***P***-value	
Complications	59 (29.6%)	25 (21.9%)	84 (26.8%)	0.138	
Pleural effusion	23 (11.6%)	10 (8.8%)	33 (10.5%)	0.440	28: conservatively
					5: drainage
Bile leak	9 (4.5%)	7 (6.1%)	16 (5.1%)	0.532	8: spontaneously resolved
					5: ERCP and stenting
					3: PTCBD
Wound infection	27 (13.6%)	12 (10.5%)	39 (12.5%)	0.433	36: antibiotics
					3: debridement
Intraabdominal hemorrhage (minor)	2 (1%)	2 (1.8%)	4 (1.3%)	0.624	PRBC transfusion
DVT	4 (2%)	3 (2.6%)	7 (2.2%)	0.703	7: LMWH
Intraabdominal collection	8 (4%)	6 (5.3%)	14 (4.5%)	0.609	8: percutaneous drainage
					6: conservatively/antibiotics
Transient hepatic failure	6 (3%)	0 (0%)	6 (1.9%)	0.09	Conservatively
Thirty-day mortality	1 (0.5%)	1 (0.9%)	2 (0.6%)		

DVT: deep vein thrombosis;

ERCP: endoscopic retrograde cholangiopancreatography;

LMWH: low molecular weight heparin;

PRBC: packed red blood cells;

PTCBD: percutaneous transhepatic biliary drainage.

Thirty-day mortality was 0.63% (2 deaths during the postoperative period). One patient died of pulmonary embolism (PE) caused by deep vein thrombosis (DVT) on postoperative day two and the other died of liver failure on postoperative day eight. The first patient, a 78-year-old obese female, developed DVT and received low molecular weight heparin subcutaneously. The second patient underwent extended right hepatectomy for a Klatskin tumor and developed liver failure immediately postoperatively.

## Discussion

Hemorrhage is one of the main factors contributing to morbidity and mortality in major liver resections. It is well known that, in cases of malignant tumors, perioperative administration of blood affects not only the disease-free survival period of the hepatectomized patient, through modulation of the immune response, but also the overall survival. An additional source of concern is the increased risk of infectious disease transmission through blood transfusion[6–11].

Conversely, the Pringle maneuver represents a valuable tool for managing intraoperative bleeding but places the patient at a high risk of liver damage due to ischemic reperfusion syndrome and other complications, such as splanchnic congestion and hemodynamic alterations due to vascular occlusion[18–24]. Intermittent occlusion, hemihepatic vascular occlusion[27], and ischemic preconditioning of the liver[28] have been used to minimize liver damage while simultaneously reducing intraoperative bleeding.

### Liver resection using CUSA® and Aquamantys® is associated with a low transfusion rate and the necessity for the Pringle maneuver is eliminated

The median blood loss reported from other specialized centers ranges from 155 ml to more than 750 ml, while the perioperative blood transfusion range is from 12.6 to 65%[29–35]. Our series has a low intraoperative blood loss (377 ml) and a low rate of blood transfusions (10.5%) that are comparable or even lower than the current published data from leading liver units as mentioned above[29–35].

The rate of blood transfusion reduces to 6.7% when the patients who underwent re-do liver resection or combined colon and hepatic resection for CRLM are excluded. Higher IBL in this subset of patients is likely to be secondary to higher blood loss either during difficult liver mobilization because of previous liver resection or during colonic resection.

This series demonstrates that parenchymal transection using CUSA® and Aquamantys® is a standardized procedure causing minimal blood loss that removes the dangers of hepatic inflow occlusion incurred with alternative techniques such as the Pringle maneuver[20–24].

CUSA® selectively destroys and aspirates parenchyma leaving vessels and biliary ducts almost intact with larger vessels and large intrahepatic bile ducts amenable to ligation or clipping. Aquamantys® delivers radiofrequency energy and causes protein denaturation. Thus, blood vessel wall collagen is shrunk, resulting in hemostasis. Hence, Aquamantys® is efficient in controlling the small bleeding vessels within the liver parenchyma. The combined use of these two devices allows almost bloodless parenchymal transection without the need for hepatic inflow occlusion and ensures an efficient overall operative time (mean parenchymal transection time is 51 minutes).

Elimination of the Pringle maneuver allows transection of the liver without the previous limitation of clamp times. This results in the opportunity for meticulous parenchymal transection and gives surgical trainees the chance to develop their skills in this procedure without the previous time pressures.

This data suggests that liver resection with the use of the combined technique of saline-linked radiofrequency ablation and ultrasonic aspiration, is a useful and efficient procedure toward bloodless liver resection without the use of vascular occlusion and ensures that liver resection becomes a comparatively safer procedure.

### Liver resection using CUSA® and Aquamantys® is associated with low postoperative morbidity and minimum mortality

Although the morbidity rate was 26.8%, it should be noted that most of these (20.1%) were minor complications.

We performed 199 major hepatectomies from which 41 were extended right or left hepatectomies: 42 of these hepatectomies were re-do hepatectomies. By removing the need for the Pringle maneuver we believe that low postoperative liver insufficiency rates (6 patients (1.9%), of which 5 were transient) can be achieved. Furthermore, removal of the Pringle maneuver eliminates concerns regarding clamp time. This time comfort in combination with the use of CUSA® dissection (which leaves biliary ducts intact) allows meticulous ligation or clipping of large intrahepatic biliary ducts with small biliary ducts dealt with using the heating effects of Aquamantys®. The above characteristics of this combined technique, we believe that is the reason of the very low rates of bile leak (5.1%) in this series.

A low 30-day mortality rate of 0.63% was achieved in this consecutive series; this is consistent with rates from current well-respected centers[29, 30, 32, 36].

Τhe greatest restriction of our study is its retrospective nature and, as such, a selection bias is a possibility. This possibility of selection bias is reduced by the fact that during the period under study all three surgeons applied the same surgical technique for liver parenchymal transection to all of their patients who underwent hepatectomy. Another restriction of this study is the absence of a comparative group. Such a group was not possible as the previously used techniques for liver parenchymal transection differed between the participating institutions.

## Conclusion

In conclusion, the limitations associated with a retrospective, single-institution study are acknowledged. However, the fairly large number of this series allows us to conclude that combined technique of saline-linked radiofrequency ablation and ultrasonic aspiration for liver resection is a safe method for both major and minor liver resections. The method is associated with decreased blood loss, reduced postoperative morbidity, and minimal mortality rates. We believe that this combined technique is comparable to other techniques and should be considered as an alternative.

## Consent

Written informed consent was obtained from the patient for the publication of this report and any accompanying images.

## Abbreviations

IBL: intraoperative blood loss
PTBL: blood loss during parenchymal transaction
CRLM: colorectal liver metastases.
